# Reciprocal regulation of hnRNP C and CELF2 through translation and transcription tunes splicing activity in T cells

**DOI:** 10.1093/nar/gkaa295

**Published:** 2020-04-27

**Authors:** Michael J Mallory, Sean P McClory, Rakesh Chatrikhi, Matthew R Gazzara, Robert J Ontiveros, Kristen W Lynch

**Affiliations:** Department of Biochemistry and Biophysics, Perelman School of Medicine, University of Pennsylvania, Philadelphia, PA 19104, USA

## Abstract

RNA binding proteins (RBPs) frequently regulate the expression of other RBPs in mammalian cells. Such cross-regulation has been proposed to be important to control networks of coordinated gene expression; however, much remains to be understood about how such networks of cross-regulation are established and what the functional consequence is of coordinated or reciprocal expression of RBPs. Here we demonstrate that the RBPs CELF2 and hnRNP C regulate the expression of each other, such that depletion of one results in reduced expression of the other. Specifically, we show that loss of hnRNP C reduces the transcription of CELF2 mRNA, while loss of CELF2 results in decreased efficiency of hnRNP C translation. We further demonstrate that this reciprocal regulation serves to fine tune the splicing patterns of many downstream target genes. Together, this work reveals new activities of hnRNP C and CELF2, provides insight into a previously unrecognized gene regulatory network, and demonstrates how cross-regulation of RBPs functions to shape the cellular transcriptome.

## INTRODUCTION

RNA binding proteins (RBPs) regulate a myriad of gene expression processes in the cell, from splicing to nuclear export to translation and RNA decay (1,2). Importantly, many RBPs also functionally regulate the expression of other RBPs through altering splicing, stability or translation (3,4). In addition, RBPs often cooperate or antagonize each other's activity on substrates (5). This complex interplay of expression and activity between RBPs is important to ultimately shape gene expression.

Alternative splicing of pre-mRNAs allows for the generation of distinct protein functions from a single gene by regulated inclusion or exclusion of particular exons or segments thereof (5). Such alternative splicing is typically controlled by RNA-binding proteins (RBPs) that associate with sites along a nascent transcript and direct the splicing machinery to sites of cleavage and ligation (5). Importantly, however, most well-studied examples of alternative splicing are regulated not by the presence or absence of a single RBP, but rather through the combinatorial activity of numerous RBPs that function in a cooperative or antagonistic manner (5). Since alternative splicing has profound impact on cellular function (6–8), understanding how RBPs functionally intersect at both the level of target activity as well as expression is important to understanding how splicing decisions are regulated.

One RBP that has been particularly linked to both alternative splicing and the regulation of other RBPs is CELF2 (9–14). CELF2 is part of the CUGBP, ELAV-Like Family (CELF) of proteins, which all contain three RNA recognition motifs (RRMs) and have been shown to regulate numerous steps in RNA processing including pre-mRNA splicing, mRNA stability and polyadenylation (12,15,16). In the case of alternative splicing, CELF2 has been shown to act as both an activator and repressor of exon inclusion, dependent on the location of its binding relative to the regulated exon (10,12,17,18). We have also shown that in Jurkat T cells, CELF2 regulates the alternative splicing of many RBPs and also regulates the expression of RBFOX2 via control of alternative polyadenylation (14,16).

CELF2 typically regulates splicing by binding to intronic UG-rich sequence elements (18). Interestingly, many of the sequence elements that have been shown biochemically to bind CELF2 also bind the RBP hnRNP C, including intronic regulatory sequences in the TRAF3, LEF1 and MKK7 genes (17–19). HnRNP C is an abundant nuclear RBP that associates both in vitro and in vivo with 4–5 consecutive uridine residues (20–22). Such poly-U stretches are common in introns and 3′ untranslated regions (UTRs). Accordingly, hnRNP C has been shown to bind to over half of protein coding genes in cells and regulates both splicing and polyadenylation (20). In particular, hnRNP C has a general role in preventing cryptic inclusion of exon-like Alu-elements, thereby maintaining the fidelity of the genome (21).

Given the similarity between the binding consensus for CELF2 (UG-rich) and hnRNP C (U-rich), it is perhaps not surprising that these proteins often co-localize on pre-mRNAs. However, the impact of this co-localization and possible functional cross-talk between CELF2 and hnRNP C has not been broadly explored. Moreover, in the few cases where cooperative function of CELF2 and hnRNP C has been studied there is no clear pattern. For example, CELF2 and hnRNP C both appear to repress use of TRAF3 exon 8 upon binding to an intronic silencer upstream from this exon (19). By contrast, both CELF2 and hnRNP C bind upstream of the second exon of the MKK7 gene (17,18), but in previous studies only knockdown of CELF2 significantly alter inclusion of this exon (17).

Here, we undertake a comprehensive analysis of the functional interplay of CELF2 and hnRNP C. We find a significant overlap of splicing events that are regulated in response to shRNA-mediated depletion of either CELF2 or hnRNP C. Further analysis revealed that this is due, at least in part, to the fact that depletion of either CELF2 or hnRNP C markedly decreased expression of the other protein. We demonstrate that hnRNP C is necessary for optimal transcription of CELF2, while CELF2 regulates the translation of hnRNP C. Finally, by reconstituting expression of one protein in the absence of the other, we show that cooperative loss of both proteins has a more robust impact on splicing than loss of either protein alone. Therefore, we conclude that the reciprocal regulation of CELF2 and hnRNP C is used by cells to achieve maximal response in cellular mRNA expression.

## MATERIALS AND METHODS

### Cell culture, knockdowns and protein expression

JSL1 Jurkat cells were grown in RPMI + 5% heat-inactivated FCS as described previously (23). Direct knockdown of CELF2 was done as described previously (9) using sequences directed to the CELF2 coding sequences (see Supplemental Figure S2). Direct knockdown of hnRNP C was done in the same manner, using a lentivirus encoded short hairpin RNAs targeting the hnRNP C 3′ UTR (see Supplemental Figure S2). Expression of cDNA CELF2, hnRNP C1 and hnRNP C2 was done as described previously (17). Stimulation of cells was done by the addition of 20ng/ml PMA. Inhibition of transcription and translation to test mRNA/protein stability was done by adding 5ug/ml actinomycin or 100 μg/ml cycloheximide, respectively, to the Jurkat culture media followed by harvesting the cells at the time points indicated.

### RASL-seq and RT-PCR analysis

Three independent integrates of the lentivirus-shRNA for both CELF2 and HNRNPC were grown along with wildtype controls and stimulated with 20 ng/ml PMA as described previously (9,23). RNA was extracted using RNA-Bee (Tel-Test). RASL-seq libraries were prepared and sequenced as described (17). Splicing events were filtered for >10 average reads across all samples. Percent spliced in (PSI) was calculated for each event by the ratio of the number of reads corresponding to the long isoform to the total reads (long and short) for the event. The change (ΔPSI) was then calculated as the difference between average PSI from the shRNA replicates and average PSI from three control replicates. Events were considered significantly changing if the absolute value for ΔPSI was greater than 10% with *P* <0.05 (unpaired Student's *t*-test). Event validation was done using radiolabeled RT-PCR as described previously (23). Primers used are listed in Supplementary Table S2. Events were considered validated if RT-PCR showed splicing changed in the same direction as indicated by RASL-seq and ΔPSI was greater than 10% with *P* < 0.05.

### qRT-PCR

Quantitative RT-PCR was done as previously described (24). PCR primers used are listed in Supplementary Table S2.

### Western blots

Western blots were done as previously described (16) using the following antibodies: hnRNP L (Abcam, Ab6106), CELF2 (University of Florida ICBR, HL1889), hnRNP C (Abcam, Ab10294), Flag (Cell Signaling Technologies, 2368S).

### 3′RACE

RACE-Ready cDNA were produced and transcript 3′ ends were identified by SMARTer RACE cDNA Amplification Kit (Clontech) according to the manufacturer's instructions and as described in (16). RACE-Ready cDNA were analyzed on agarose gels. Gene specific forward primer used for amplification of RACE-Ready cDNA is listed in Supplementary Table S2.

### Polysome analysis

Polysome analysis of Jurkat cells was done based on methods described previously (25). In brief, 6 × 10^7^ cells were either treated with cycloheximide (CHX) 100 μg/ml for 30 min or left untreated. Cells were then pelleted and rinsed with ice-cold PBS containing 100 μg/ml CHX. Cell pellets were resuspended in 1 ml of ice-cold lysis buffer (10 mM HEPES, pH 7.4, 5 mM MgCl_2_, 100 mM KCl, 1% Triton X-100, 2 mM DTT, 100 μg/ml CHX, 1× Complete EDTA-free protease inhibitor cocktail (Roche) and 400 U/ml RNAsin (Promega)) and passed through QIAShredder microbead columns (Qiagen). 700 μl of lysate was loaded onto 10–50% sucrose gradients buffered with 10 mM HEPES, pH 7.4, 5 mM MgCl_2_, 100 mM KCl, 2 mM DTT, 100 μg/ml CHX, 1× Complete EDTA-free protease inhibitor cocktail and 40 U/ml RNAsin and centrifuged in a SW-40 rotor at 38 000 RPM for 135 min. Gradients were fractionated as described (25). 1 fmol of T7 transcribed influenza-A M1-RNA was added to each fraction as a control and RNA extracted using RNA-Bee (Tel-Test).

## RESULTS

### HnRNP C and CELF2 regulate a common set of splicing events

**Figure 1.**
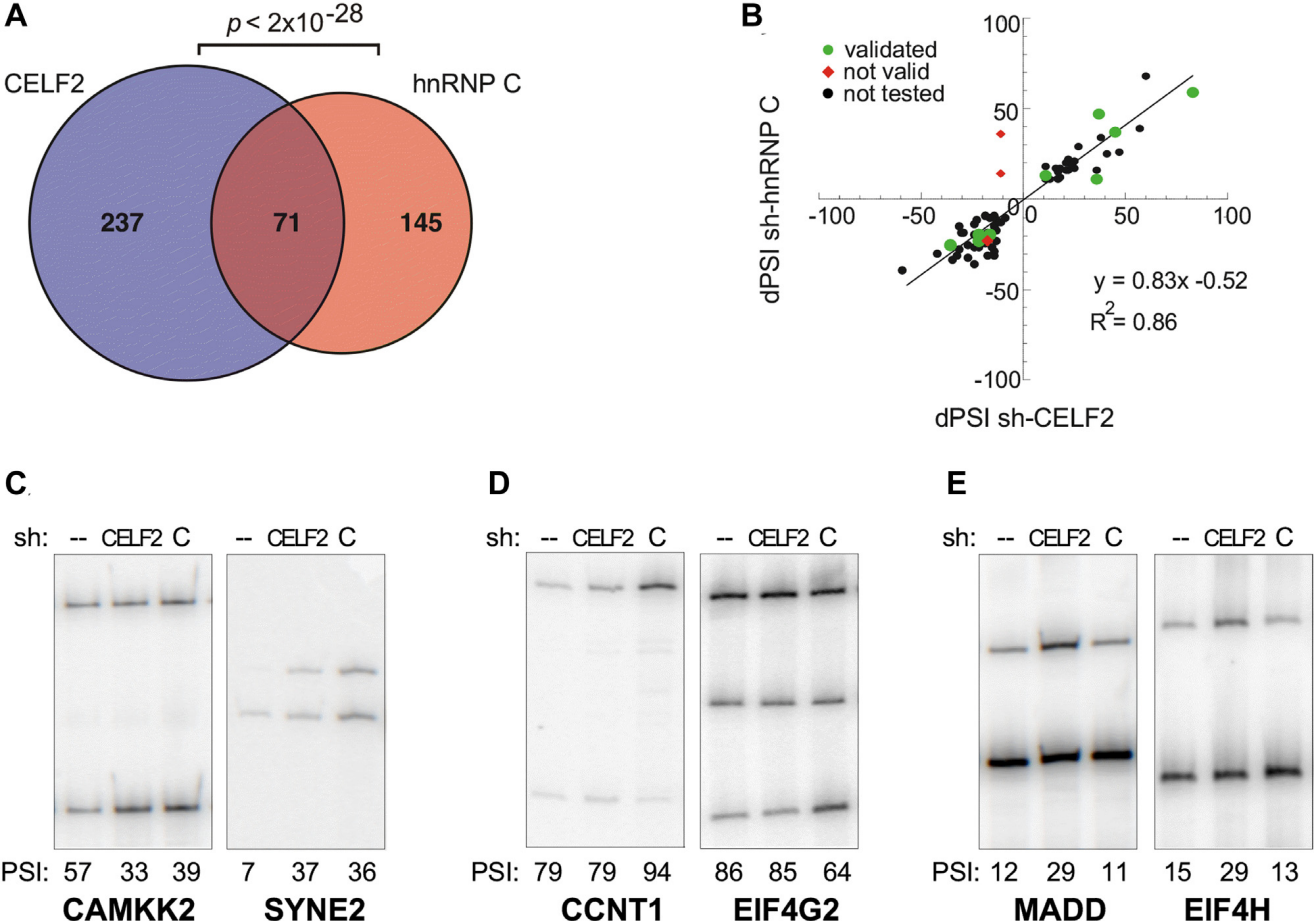
CELF2 and hnRNP C regulate a highly overlapping set of splicing events. (**A**) Overlap of events sensitive (|ΔPSI| > 10, *P*< 0.05) to either knockdown by shCELF2 or sh-hnRNPC in PMA stimulated Jurkat cells. Significance of overlap was calculated using a 2-tailed hypergeometric test. (**B**) Correlation of ΔPSI of the 71 splicing events sensitive to both shCELF2 and sh-hnRNP C in PMA stimulated Jurkat cells. Splicing events validated by RT-PCR are shown in green, while events that failed to validate by RT-PCR are shown as red diamonds. (C–E) Representative RT-PCR analysis of the splicing of genes predicted by RASL-Seq to be regulated by (**C**) both hnRNP C and CELF2, (**D**) hnRNP C only or (**E**) CELF2 only. Splicing is quantified by the percent of variable exon inclusion (PSI). Values and standard deviation from at least three biologically independent experiments is shown below in Figure 5.

The previous reports demonstrating binding of CELF2 and hnRNP C to overlapping splicing regulatory sequences suggest these proteins may coordinately regulate a similar set of genes. To test this prediction, we compared the impact of depletion of CELF2 or hnRNP C in Jurkat T cell on the splicing of ∼5000 known alternative exons using the RASL-Seq platform (26). Indeed, in stimulated cells in which expression of both CELF2 and hnRNP C are normally robust, we find a high degree of overlap between exons that exhibit altered splicing upon shRNA-mediated knockdown of CELF2 versus shRNA-mediated knockdown of hnRNP C (23% of CELF2-responsive or 33% of hnRNP C-responsive, *P*< 2e–28) (Figure 1A, Supplemental Table S1). This overlap is much more than that observed between other pairwise comparison of RBPs in Jurkat cells (Supplemental Figure S1A). Even more strikingly, for those exons responsive to both hnRNP C and CELF2, the direction and extent of impact (enhancement versus repression) is nearly identical (*R*^2^ = 0.86 with a slope of 0.83, Figure 1B). The only two splicing events that were predicted by the RASL platform to exhibit opposite changes upon depletion of CELF2 versus hnRNP C were shown to be false predictions by subsequent RT-PCR analysis (Figure 1B, ‘not valid’). By contrast, the vast majority of predicted co-regulated events were validated by RT-PCR (Figure 1B, C, Supplemental Table S1). We also find that many of the splicing changes that initially appear to be specific for CELF2 or hnRNP C (Figure 1A) actually trend in the same direction upon depletion of the other proteins (Supplementary Table S1). However, we do find 37 and 45 splicing events that are truly specific for hnRNP C or CELF2, respectively (Supplementary Table S1), several of which were confirmed by RT-PCR (Figure 1D, E). A similar trend is also observed in unstimulated Jurkat cells (Supplemental Figure S1B, C), although CELF2 is only weakly expressed under these conditions and thus the impact of depletion of CELF2 is less.

### The expression of hnRNP C and CELF2 proteins are co-regulated

**Figure 2.**
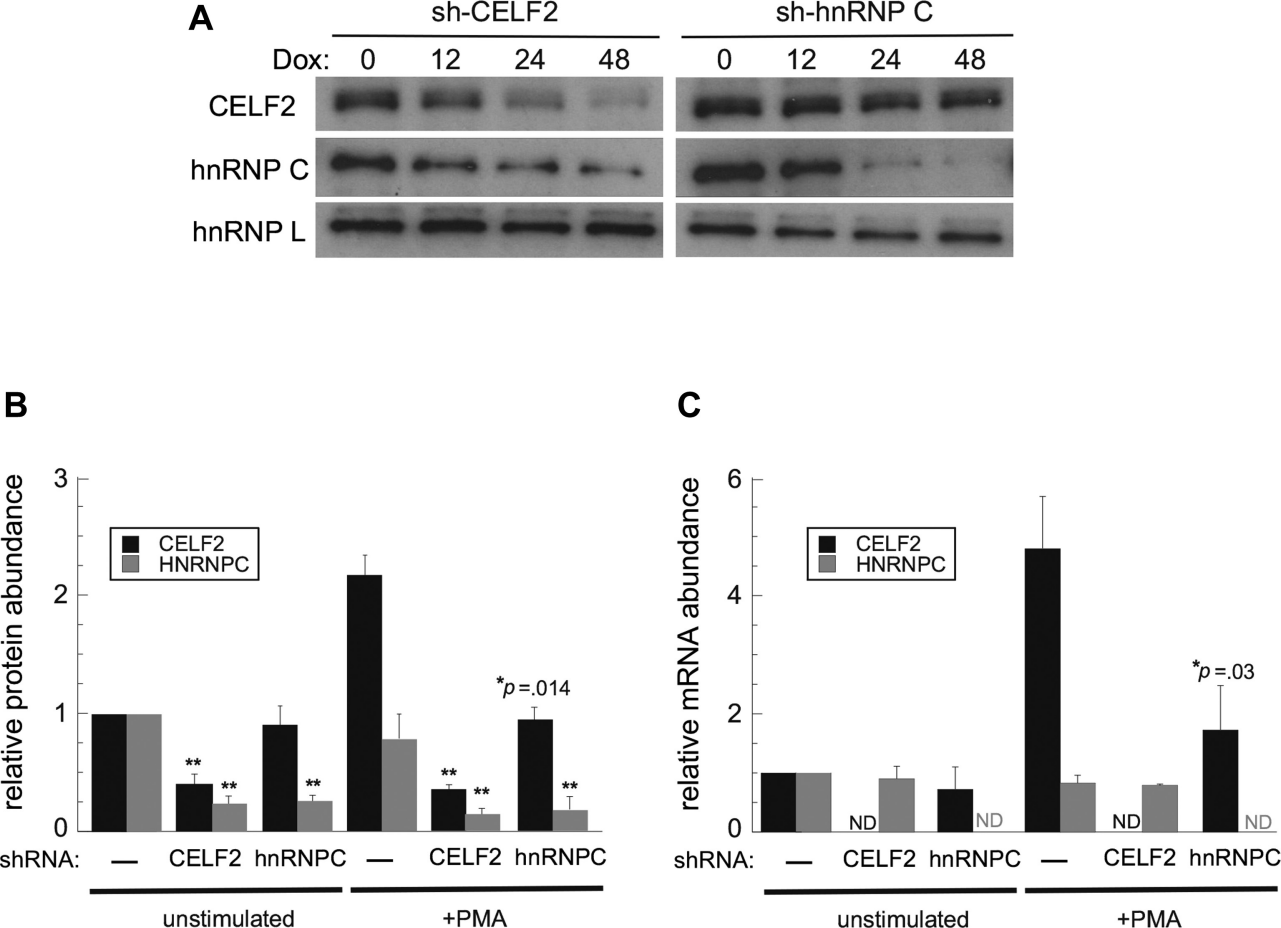
HnRNP C promotes CELF2 mRNA expression while CELF2 promotes hnRNP C protein expression. (**A**) Western blot of CELF2 and hnRNP C upon shRNA-mediated knock-down of either protein in PMA-stimulated Jurkat cells. HnRNP L is used as a loading control. (**B**) Quantification of protein expression 48 h after knock-down, as shown in representative blots in panel A and Supplemental Figure S2. Values are from three biologically independent experiments and normalized to the expression of each protein in unstimulated wildtype cells. (**C**) Quantification of CELF2 and hnRNP C mRNA by qPCR from unstimulated or PMA-stimulated wildtype Jurkat cells compared to those depleted of CELF2 or hnRNP C for 48 hours. ND indicates mRNA was below the level of detection. Double asterisk indicates *P*< 0.01.

While the high degree of co-regulation of splicing by CELF2 and hnRNP C is consistent with these proteins exhibiting overlapping binding sites on pre-mRNAs, another potential explanation is that depletion of one protein impacts the expression of the other. To test this, we performed western blot and qPCR analysis of protein and RNA expression, respectively. Strikingly, we indeed observe that depletion of CELF2 in stimulated cells results in a ∼5-fold reduction in hnRNP C protein (Figure 2A, B), with little impact on hnRNP C mRNA (Figure 2C, gray bars in – versus CELF2 shRNA). A similar trend is observed in unstimulated cells, however, as the expression of CELF2 is naturally low in unstimulated cells its depletion has a more limited effect (Figure 2B, Supplemental Figure S2A). Conversely, depletion of hnRNP C has a modest (∼2-fold) effect on CELF2 protein (Figure 2A, B) that is mirrored by a ∼2-fold decrease in CELF2 mRNA (Figure 2C, black bars). Importantly, we observe a similar decrease in hnRNP C protein with two independent CELF2 targeting shRNAs and visa versa, and there is little complementarity between each shRNA and the other non-targeted mRNA (Supplemental Figure S2B–D). Therefore, we conclude that any impact of depletion of CELF2 or hnRNP C on the converse protein is not due to off-targeting effects of shRNAs, but rather to cross-regulation between these proteins.

### HnRNP C regulates the transcription of CELF2 mRNA

We first explored the mechanism for the apparent regulation of CELF2 by hnRNP C. We have previously shown that CELF2 mRNA is subject to both alternative splicing and alternative polyadenylation (APA) (16). Knockdown of hnRNP C for 24 hours has no impact on the pattern of CELF2 alternative splicing or CELF2 APA (Supplemental Figure S3). We did not assess later time points as CELF2 regulates its own splicing and APA, thus once CELF2 protein starts to decrease we anticipate autoregulatory impact on its own transcript. Furthermore, since neither alternative splicing nor APA impacts the level of CELF2 mRNA (16), it is unlikely that either process is the mechanism by which loss of hnRNP C leads to a reduction in CELF2 mRNA.

**Figure 3.**
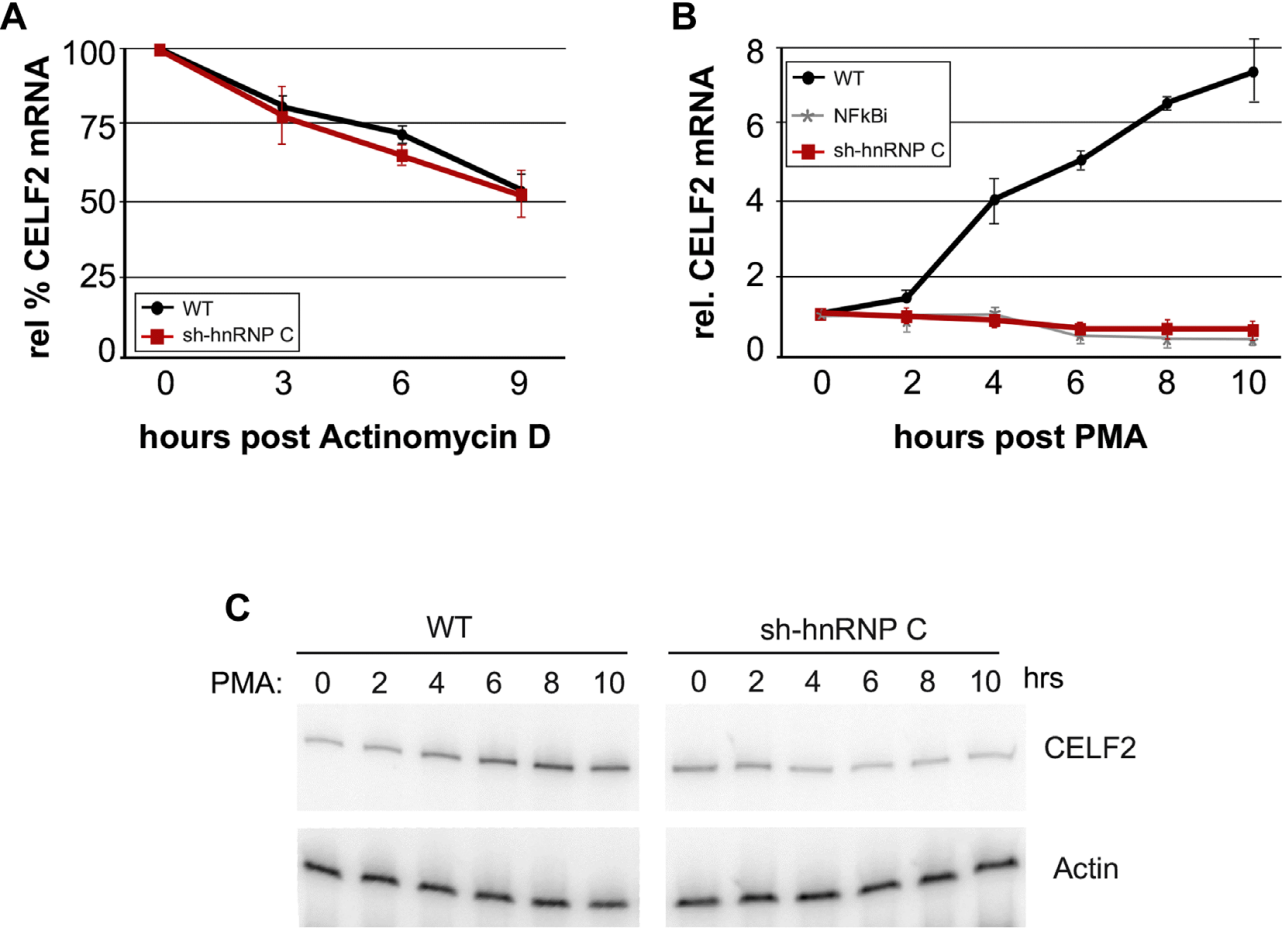
HnRNP C promotes transcription of CELF2. (**A**) Quantification of CELF2 mRNA following inhibition of transcription by actinomycin D in wildtype Jurkat cells (WT) versus those expressing shRNA against hnRNP C (sh-hnRNP C) is shown. CELF2 levels were measured by RT-PCR and normalized to actin. (**B**) CELF2 mRNA expression, as measured by RT-PCR and normalized to actin, following PMA treatment of wildtype Jurkat cells (WT) versus those expressing shRNA against hnRNP C (sh-hnRNP C). Treatment of Jurkat cells with an inhibitor to NF-κB (NFκBi) was previously shown to block the transcriptional induction induced by PMA (9). For both panels A and B, values are from at least 3 biologically independent experiments. Error bars represent standard deviation. (**C**) A representative gel used for quantification in panel B.

We have previously observed that CELF2 mRNA is subject to regulated transcription and stabilization (9,17). We first assessed whether depletion of hnRNP C impacts the stability of CELF2 using actinomycin D to block transcription, but observed no change in CELF2 mRNA half-life (Figure 3A). By contrast, we do observe a loss of the NFkB-induced transcription of CELF2 (Figure 3B, C) that we have reported previously (9). The impact of hnRNP C on the transcription of CELF2 mRNA, which is responsible for roughly half of the increase in CELF2 expression in PMA stimulated cells, is consistent with the partial decrease in CELF2 mRNA and protein observed upon loss of hnRNP C. The mechanism by which hnRNP C may contribute to the transcription of CELF2 is not fully clear but is consistent with the fact that hnRNP C has been linked to NFkB signaling (19).

### CELF2 regulates the translation of hnRNP C mRNA

**Figure 4.**
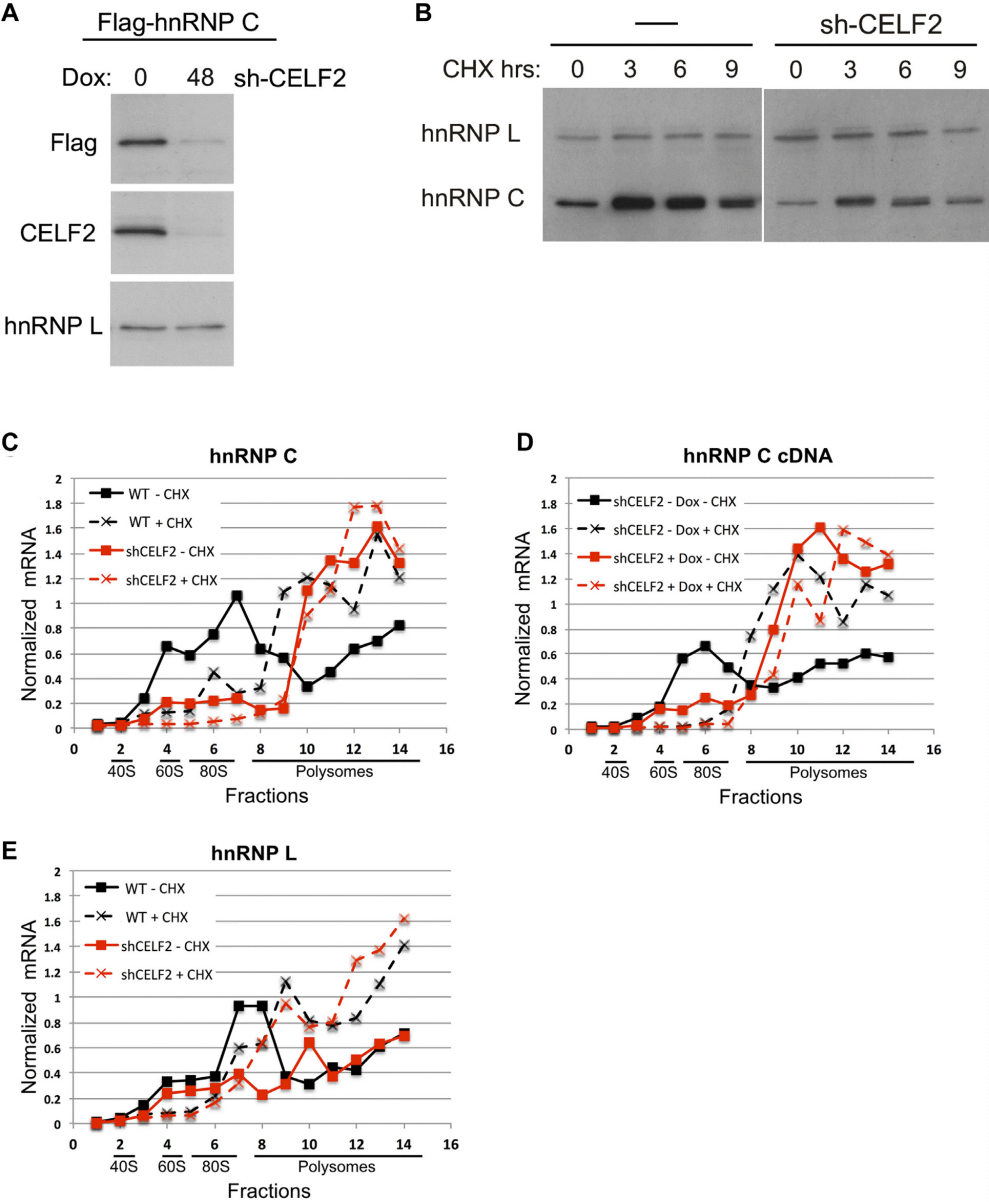
CELF2 regulates translation of hnRNP C. (**A**) Western blot of Flag-tagged hnRNP C (C1) expressed from a transfected cDNA in the absence or presence of Doxycycline induced expression of shRNA against CELF2. Depletion of CELF2 is also confirmed by western blot. hnRNP L is a loading control. (**B**) Expression of hnRNP C or hnRNP L protein, by western blot, following inhibition of translation by treatment with cycloheximide (CHX). (C–E) Quantification of the indicated messages in RNA extracted from the indicated fractions following sucrose gradient separation of polysomes from wildtype (WT) or CELF2 shRNA (shCELF2) expressing Jurkat cells harvested in the absence (–CHX) or presence (+CHX) of cycloheximide. mRNAs quantified are (**C**) endogenous hnRNP C, (**D**) Flag-tagged hnRNP C cDNA, (**E**) hnRNP L. In the case of hnRNP C cDNA, the control cells did contain the shRNA vector against CELF2, but the expression of this hairpin was not induced by doxycycline (–Dox). All mRNA levels were quantified by RT-PCR and normalized to RT-PCR signal from exogenous influenza viral M1 RNA spiked into fractions prior to RNA isolation. Results from replicate experiments and overall polysome profiles are shown in Supplemental Figure S6.

Having determined that hnRNP C contributes to the transcriptional expression of CELF2, we next sought to understand how CELF2 regulates the expression of hnRNP C. Depletion of CELF2 reduces hnRNP C protein without a corresponding change in hnRNP C mRNA levels (Figure 2C) or splicing (Supplemental Figure S4). In addition, depletion of CELF2 also causes a reduction in Flag-tagged hnRNP C expressed from a cDNA construct (Figure 4A, Supplemental Figure S5). Therefore, we considered mechanisms that specifically control protein levels, namely protein stability and translation. While we observe some stabilization and a slight mobility shift of hnRNP C upon treatment with the translation blocker cycloheximide, this occurs in both the presence and absence of CELF2 depletion (Figure 4B), with the only difference being the reduced steady-state levels of hnRNP C in the presence of CELF2 depletion, as observed above (Figure 2). Therefore, we conclude that CELF2 does not have a significant impact on hnRNP C protein stability.

In contrast to the lack of difference in protein stability, we do observe a CELF2-dependent difference in the association of ribosomes with hnRNP C mRNA (Figure 4C, Supplemental Figure S6A). In wildtype cells, hnRNP C mRNA is enriched in high density polysome fractions following a brief treatment with cycloheximide (WT +CHX) but is depleted from polysomes in the absence of cycloheximide (WT –CHX), as is typical of efficiently translating messages (27). Overall polysomes are also depleted in the absence of cycloheximide (Supplemental Figure S6B). However, upon expression of shRNA against CELF2, hnRNP C mRNA is retained in polysomes even in the absence of cycloheximide (Figure 4C, shCELF2 –CHX). Such a cycloheximide-independent association of mRNAs with polysomes is considered an indication of poor efficiency of translation elongation (27,28). Notably, we observe a similar cycloheximide-independent ribosome profile for hnRNP C cDNA (Figure 4D), but not for the heterologous hnRNP, hnRNP L, whose expression is not impacted by CELF2 (Figure 4E). In sum, we conclude that depletion of CELF2 reduces translation elongation efficiency in a transcript-specific manner, specifically including the hnRNP C mRNA. A detailed investigation of the mechanism by which CELF2 depletion impacts translation is beyond the scope of the present study, but several hypotheses are described below (see Discussion).

### Coordinated expression of CELF2 and hnRNP C is important for optimal splicing regulation

Having determined the mechanisms by which CELF2 and hnRNP C regulate the expression of each other, we next investigated the physiologic implications of this regulation. One question is whether CELF2 and hnRNP C are coordinately expressed under changing cellular conditions. This question is complicated given that CELF2 and hnRNP C, like most RBPs, are regulated at many different levels (9,16,19) and changes in cell signaling or cell fate can induce multiple regulatory programs. For example, the transcription of hnRNP C decreases upon activation of Jurkat cells in a non-CELF2 dependent manner (17,24); therefore, although CELF2 protein levels increase upon T cell activation due to increased protein transcription and stability, the level of hnRNP C protein remains roughly unchanged or even decreases slightly (see Supplemental Figures S2 and S7). However, the bidirectional regulation of CELF2 and hnRNP C appears to be an important factor in the ultimate expression of these proteins upon activation, as CELF2 does not increase upon stimulation in the absence of hnRNP C (Supplemental Figure S2A) and in the complete absence of CELF2, hnRNP C decreases to almost undetectable levels upon stimulation (Supplemental Figure S7). Moreover, proteomic (13,29) and transcriptomic (30) analysis of mouse heart and muscle tissue also demonstrates a parallel reduction of CELF2 and hnRNP C during postnatal development (Supplemental Figure S7). Therefore, while cross-regulation of CELF2 and hnRNP C is not the only determinant of expression of these two proteins, there is evidence that coordinated expression tunes the levels of these proteins under physiologic conditions.

**Figure 5.**
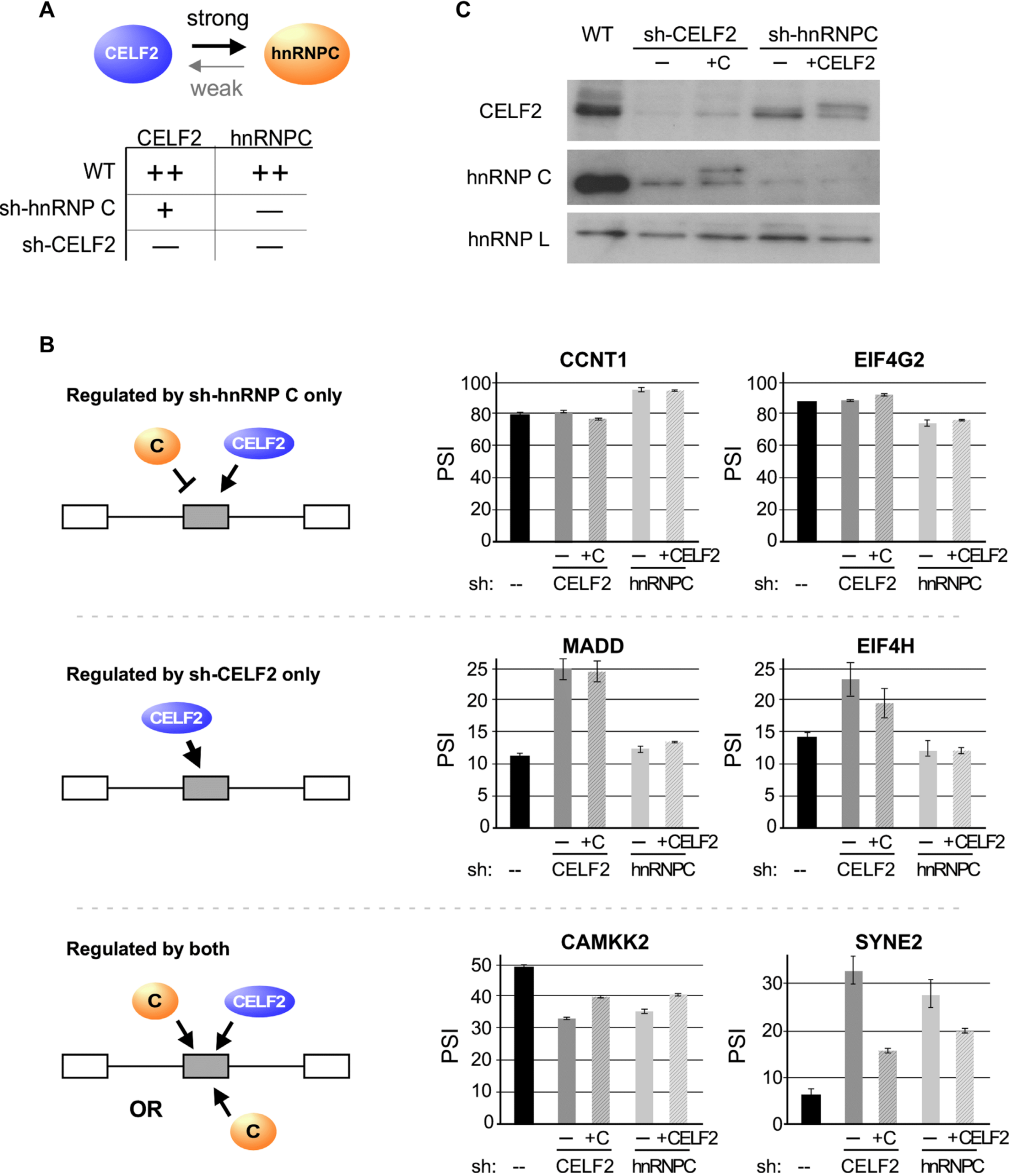
Coordinated expression of CELF2 and hnRNP C fine-tune splicing outcomes of target genes. (**A**) Schematic and summary of the consequence of expression of shRNA against CELF2 (shC2) or hnRNP C (shC) on the expression of both proteins, as listed on the top of the columns. ++, + and − indicate high, medium and low expression. (**B**) Left: Potential models of action of CELF2 and hnRNP C on splicing targets that account for both the coordinated regulation observed between these proteins and the subset of target genes that are impacted by depletion of only one or the other protein. See main text for detailed description of models. Right: Quantification of RT-PCR analysis of several target genes upon partial rescue of CELF2 or hnRNP C expression in the knock-down of the other gene. Values are derived from at least three biologically independent experiments. Error bars represent standard deviation. (**C**) Western blot of CELF2 and hnRNP C expression in cells used for RT-PCR analysis in panel B. +C and +C2 indicate the overexpression of hnRNP C or CELF2, respectively, from Flag-tagged cDNA vectors. The mobility of the cDNA-expressed proteins is different from the endogenous due to the Flag tag. HnRNP L is used as a loading control.

Finally, we sought to understand the functional consequence of co-regulation of CELF2 and hnRNP C on the splicing of target genes. In particular, given the fact that depletion of either CELF2 and hnRNP C partially or fully reduces the expression of the other (Figure 5A) we asked why splicing of some target genes appear to be specifically impacted only in the case of one of the hairpins. We hypothesized that splicing events that are only altered in the presence of sh-hnRNP C may be cases in which CELF2 and hnRNP C have direct antagonistic impact, such that preferential reduction of hnRNP C with the sh-hnRNP C hairpin shifts the balance of regulation toward CELF2, while reduction of both proteins in the case of the sh-CELF2 hairpin would restore the balance to that of the wildtype situation (Figure 5B, top left). Consistent with this, we observe a strong signature for hnRNP C binding around these exons using CLIP data from ENCODE (Supplemental Figure S8). While we observe less binding of CELF2 around these sh-hnRNP C-specific exons, it is possible that CELF2 exerts its effect indirectly, such as through RBFOX2 as we have shown previously (14). Regardless, this model predicts that forced expression of CELF2 would have little impact on the sh-hnRNP C induced splicing change for these events, as CELF2 is already active. Indeed, this is what we observe when we used cDNA overexpression to increase Flag-tagged CELF2 expression in the presence of the hnRNP C hairpin (Figure 5B, top right, CCNT1, EIF4G2 and Figure 5C). By contrast, those events that are specifically altered in the presence of the sh-CELF2 hairpin are likely ones in which hnRNP C has effect and CELF2 repression is saturating, such that the partial depletion observed in the presence of the sh-hnRNP C hairpin is not sufficient to yield an effect (Figure 5B, middle left). Again, this is consistent with the CLIP data that reveals a strong signature for CELF2 binding around these sh-CELF2-specific exons, but little evidence for hnRNP C binding (Supplemental Figure S8). Moreover, re-expression of Flag-tagged hnRNP C had no impact on genes regulated only in the presence of the CELF2 shRNA (Figure 5B, middle right, MADD, EIF4H and Figure 5C), while overexpression of Flag-tagged CELF2 in wildtype cells was sufficient in many cases to induce changes in splicing (Supplemental Figure S9).

Lastly, we wanted to determine whether those splicing events that are sensitive to knock down of either CELF2 or hnRNP C (Figure 5B, bottom) are directly regulated by both CELF2 and hnRNP C or, alternatively, are only controlled by hnRNP C which is efficiently reduced upon depletion of CELF2. Importantly, the above-mentioned CLIP data shows strong evidence for position-specific regulation via direct binding of CELF2, at least for those exons enhanced by CELF2, which are the majority (Supplemental Figure S8). There is also a clear signature for binding of hnRNP C around these exons (Supplemental Figure S8). Consistently, using the cDNA overexpression strategy (Figure 5C) we find that re-expression of Flag-tagged CELF2 or hnRNP C individually abrogates some, but not all, of the splicing effect of genes regulated by both the CELF2 and hnRNP C hairpins (Figure 5B, bottom right, CAMKK2, SYNE2). This partial rescue, together with the binding data, strongly supports a model of cooperative regulation of splicing by hnRNP C and CELF2, such that their coordinate expression results in maximal impact on exon inclusion. In sum, while we cannot discount a variety of other models by which hnRNP C and CELF2 regulation may impact splicing, our data supports the notion that reciprocal regulation of hnRNP C and CELF2 is used by cells to tune splicing patterns across a broad range of genes.

## DISCUSSION

HnRNP C and CELF2 are both multifunctional RNA binding proteins that have been implicated in controlling many steps of RNA processing and gene expression. While both of these proteins have been studied extensively in isolation, we show here that they also regulate a highly overlapping set of splicing events. Some of this functional overlap in splicing regulation is likely due to overlap of substrate binding, as has been demonstrated in other studies (17–19). However, we also find here that the expression of hnRNP C and CELF2 proteins are induced by each other, such that a decrease in one protein results in a partial to significant decrease in the other. We note that at least two previous studies, including one of our own, have failed to show reciprocal regulation of hnRNP C and CELF2 (17,19); however, in both of these cases the efficiency of knockdown was less than we achieve here. Importantly, we demonstrate here that with robust depletion, the coordination of hnRNP C and CELF2 expression occurs through reciprocal regulation in which hnRNP C controls the transcription of CELF2 mRNA, while CELF2 is necessary for efficient translation of hnRNP C protein. Taken together this study, therefore, adds to our understanding of the functional interplay of RNA binding proteins, and also identifies previously unknown activities of both hnRNP C and CELF2.

CELF2 mRNA is induced upon stimulation of Jurkat cells with PMA, due to an early burst of NF-κB-dependent transcription followed by a JNK-mediated increased in mRNA stability starting 24 h after stimulation. Our data demonstrate that in the absence of hnRNP C, NF-κB dependent transcription of CELF2 fails to occur. Previous studies have implicated hnRNP C as a regulator of splicing (20,31) and 3′ end processing (32) as well as mRNA stability (33) and packaging (34). To our knowledge, hnRNP C has not previously been shown to regulate transcription. However, hnRNP C has been shown to control NF-κB activity in Jurkat cells through regulating the splicing of TRAF3 (19). Therefore, we conclude that hnRNP C regulates the transcription of CELF2 indirectly through the splicing of TRAF3 and subsequent control of NF-kB.

Like, hnRNP C, CELF2 has also been shown to regulate a myriad of RNA processing steps including splicing, polyadenylation, editing and stability (15). A few studies have suggested that CELF2 may repress translation of two mRNAs through binding to the 3′UTR and competitively inhibiting association of the translation regulator HuR (15,35,36). Although CELF2 is primarily nuclear in Jurkat cells (10), it is possible that the small cytoplasmic pool of CELF2 may similarly enhance hnRNP C translation by inhibiting binding of a translation repressor; however this would have to occur within the coding sequence itself as loss of CELF2 reduces translation of the hnRNP C cDNA as robustly as it impacts the endogenous hnRNP C message. Alternatively, CELF2 may control hnRNP C translation indirectly. In such a model CELF2 would regulate the expression of a translation elongation factor through splicing, polyadenylation or stability. Regardless of a direct or indirect mode of action, we emphasize that the impact of CELF2 on translation is substrate-specific, rather than a general impact on the translation machinery, as we observe no change in the translation of hnRNP L or other control genes tested. Such a substrate-specific effect suggests either the activity of a sequence-specific RBP or an effect on specific codons. The precise mechanism by which CELF2 regulates the translation of hnRNP C, therefore, will be of great interest for future studies as a model for specific modulation of translation.

Finally, beyond providing new insight into the cellular activities of hnRNP C and CELF2, this study adds to our growing appreciation of the complex networks of RBPs that control gene expression in human cells. Auto-regulation and cross-regulation of RBPs has been extensively documented in the literature (3,14,37). This includes frequent reciprocal regulation between paralogs such as hnRNP L and hnRNP L-like (38) or PTBP1 and PTBP2 (39–41), which results in the flipping of activities on common binding sites. Notably, CELF2 and CELF1 do not show similar reciprocal regulation, at least in Jurkat T cells, perhaps because their subcellular localization is distinct (10). However, we have previously reported cross-regulation of RBFOX2 by CELF2 (14). Although RBFOX2 and CELF2 bind highly distinct sequences, these proteins do both bind an overlapping set of target genes such that the balance of expression between RBFOX2 and CELF2 does have a large impact on cellular splicing patterns (14).

Here, we show an alternate pattern of cross-regulation in which increased expression of either CELF2 or hnRNP C promotes the expression of the other protein. This cooperative expression is perhaps surprising in that the preferred binding motif for these proteins is similar and they also share many target genes in common ((17–19) and this study). However, unlike in cases such as PTBP1/PTBP2, in which the isoforms have antagonistic activities (39–41), we find evidence for wide-spread cooperative activity between hnRNP C and CELF2, such that their cooperative expression promotes splicing patterns to an extent that is greater than either protein alone. Therefore, this cooperative regulation represents a further mechanism by which RBPs tune each other's expression to ultimately achieve maximal control over cellular gene expression.

## Supplementary Material

gkaa295_Supplemental_Files

## References

[1] Zhong X.Y., Wang P., Han J., Rosenfeld M.G., Fu X.D. SR proteins in vertical integration of gene expression from transcription to RNA processing to translation. Mol. Cell. 2009; 35:1–10. 19595711 10.1016/j.molcel.2009.06.016 PMC2744344

[2] Hentze M.W., Castello A., Schwarzl T., Preiss T. A brave new world of RNA-binding proteins. Nat. Rev. 2018; 19:327–341. 10.1038/nrm.2017.130 29339797

[3] Huelga S.C., Vu A.Q., Arnold J.D., Liang T.Y., Liu P.P., Yan B.Y., Donohue J.P., Shiue L., Hoon S., Brenner S. et al. Integrative genome-wide analysis reveals cooperative regulation of alternative splicing by hnRNP proteins. Cell Rep. 2012; 1:167–178. 22574288 10.1016/j.celrep.2012.02.001 PMC3345519

[4] Ciafre S.A., Galardi S. microRNAs and RNA-binding proteins: a complex network of interactions and reciprocal regulations in cancer. RNA Biol. 2013; 10:935–942. 23696003 10.4161/rna.24641 PMC4111733

[5] Fu X.D., Ares M. Jr Context-dependent control of alternative splicing by RNA-binding proteins. Nat. Rev. Genet. 2014; 15:689–701. 25112293 10.1038/nrg3778 PMC4440546

[6] Martinez N.M., Lynch K.W. Control of alternative splicing in immune responses: many regulators, many predictions, much still to learn. Immunol. Rev. 2013; 253:216–236. 23550649 10.1111/imr.12047 PMC3621013

[7] Black D.L., Grabowski P.J. Alternative pre-mRNA splicing and neuronal function. Prog. Mol. Subcell. Biol. 2003; 31:187–216. 12494767 10.1007/978-3-662-09728-1_7

[8] Cooper T.A., Wan L., Dreyfuss G. RNA and disease. Cell. 2009; 136:777–793. 19239895 10.1016/j.cell.2009.02.011 PMC2866189

[9] Mallory M.J., Allon S.J., Qiu J., Gazzara M.R., Tapescu I., Martinez N.M., Fu X.D., Lynch K.W. Induced transcription and stability of CELF2 mRNA drives widespread alternative splicing during T-cell signaling. PNAS. 2015; 112:E2139–E2148. 25870297 10.1073/pnas.1423695112 PMC4418860

[10] Mallory M.J., Jackson J., Weber B., Chi A., Heyd F., Lynch K.W. Signal- and development-dependent alternative splicing of LEF1 in T cells is controlled by CELF2. Mol. Cell. Biol. 2011; 31:2184–2195. 21444716 10.1128/MCB.05170-11 PMC3133246

[11] Zhang W., Liu H., Han K., Grabowski P.J. Region-specific alternative splicing in the nervous system: implications for regulation by the RNA-binding protein NAPOR. RNA. 2002; 8:671–685. 12022233 10.1017/s1355838202027036 PMC1370287

[12] Ladd A.N., Charlet N., Cooper T.A. The CELF family of RNA binding proteins is implicated in cell-specific and developmentally regulated alternative splicing. Mol. Cell. Biol. 2001; 21:1285–1296. 11158314 10.1128/MCB.21.4.1285-1296.2001 PMC99581

[13] Kalsotra A., Xiao X., Ward A.J., Castle J.C., Johnson J.M., Burge C.B., Cooper T.A. A postnatal switch of CELF and MBNL proteins reprograms alternative splicing in the developing heart. PNAS. 2008; 105:20333–20338. 19075228 10.1073/pnas.0809045105 PMC2629332

[14] Gazzara M.R., Mallory M.J., Roytenberg R., Lindberg J.P., Jha A., Lynch K.W., Barash Y. Ancient antagonism between CELF and RBFOX families tunes mRNA splicing outcomes. Genome Res. 2017; 27:1360–1370. 28512194 10.1101/gr.220517.117 PMC5538552

[15] Dasgupta T., Ladd A.N. The importance of CELF control: molecular and biological roles of the CUG-BP, Elav-like family of RNA-binding proteins. Wiley Interdiscip. Rev. RNA. 2011; 3:104–121. 22180311 10.1002/wrna.107 PMC3243963

[16] Chatrikhi R., Mallory M.J., Gazzara M.R., Agosto L.M., Zhu W.S., Litterman A.J., Ansel K.M., Lynch K.W. RNA binding protein CELF2 regulates Signal-Induced alternative polyadenylation by competing with enhancers of the polyadenylation machinery. Cell Rep. 2019; 28:2795–2806. 31509743 10.1016/j.celrep.2019.08.022 PMC6752737

[17] Martinez N.M., Agosto L., Qiu J., Mallory M.J., Gazzara M.R., Barash Y., Fu X.D., Lynch K.W. Widespread JNK-dependent alternative splicing induces a positive feedback loop through CELF2-mediated regulation of MKK7 during T-cell activation. Genes Dev. 2015; 29:2054–2066. 26443849 10.1101/gad.267245.115 PMC4604346

[18] Ajith S., Gazzara M.R., Cole B.S., Shankarling G., Martinez N.M., Mallory M.J., Lynch K.W. Position-dependent activity of CELF2 in the regulation of splicing and implications for signal-responsive regulation in T cells. RNA Biol. 2016; 13:569–581. 27096301 10.1080/15476286.2016.1176663 PMC4962813

[19] Schultz A.S., Preussner M., Bunse M., Karni R., Heyd F. Activation-dependent TRAF3 exon 8 alternative splicing is controlled by CELF2 and hnRNP C binding to an upstream intronic element. Mol. Cell. Biol. 2017; 37:e00488-16. 28031331 10.1128/MCB.00488-16 PMC5359431

[20] Zarnack K., Konig J., Tajnik M., Martincorena I., Eustermann S., Stevant I., Reyes A., Anders S., Luscombe N.M., Ule J. Direct competition between hnRNP C and U2AF65 protects the transcriptome from the exonization of Alu elements. Cell. 2013; 152:453–466. 23374342 10.1016/j.cell.2012.12.023 PMC3629564

[21] Attig J., Ruiz de Los Mozos I., Haberman N., Wang Z., Emmett W., Zarnack K., Konig J., Ule J. Splicing repression allows the gradual emergence of new Alu-exons in primate evolution. Elife. 2016; 5:e19545. 27861119 10.7554/eLife.19545 PMC5115870

[22] Wan L., Kim J.K., Pollard V.W., Dreyfuss G. Mutational definition of RNA-binding and protein-protein interaction domains of heterogeneous nuclear RNP C1. J. Biol. Chem. 2001; 276:7681–7688. 11113151 10.1074/jbc.M010207200

[23] Lynch K.W., Weiss A. A model system for the activation-induced alternative-splicing of CD45 implicates protein kinase C and Ras. Mol. Cell. Biol. 2000; 20:70–80. 10594010 10.1128/mcb.20.1.70-80.2000 PMC85051

[24] Cole B.S., Tapescu I., Allon S.J., Mallory M.J., Qiu J., Lake R.J., Fan H.Y., Fu X.D., Lynch K.W. Global analysis of physical and functional RNA targets of hnRNP L reveals distinct sequence and epigenetic features of repressed and enhanced exons. RNA. 2015; 21:2053–2066. 26437669 10.1261/rna.052969.115 PMC4647460

[25] Ji X., Kong J., Liebhaber S.A. In vivo association of the stability control protein alphaCP with actively translating mRNAs. Mol. Cell. Biol. 2003; 23:899–907. 12529395 10.1128/MCB.23.3.899-907.2003 PMC140719

[26] Li H., Qiu J., Fu X.D. RASL-seq for massively parallel and quantitative analysis of gene expression. Curr Protoc Mol Biol. 2012; doi:10.1002/0471142727.mb0413s98. 10.1002/0471142727.mb0413s98 PMC3325489 22470064

[27] Saini P., Eyler D.E., Green R., Dever T.E. Hypusine-containing protein eIF5A promotes translation elongation. Nature. 2009; 459:118–121. 19424157 10.1038/nature08034 PMC3140696

[28] Ortiz P.A., Kinzy T.G. Dominant-negative mutant phenotypes and the regulation of translation elongation factor 2 levels in yeast. Nucleic Acids Res. 2005; 33:5740–5748. 16214807 10.1093/nar/gki882 PMC1253829

[29] Talman V., Teppo J., Poho P., Movahedi P., Vaikkinen A., Karhu S.T., Trost K., Suvitaival T., Heikkonen J., Pahikkala T. et al. Molecular atlas of postnatal mouse heart development. J. Am. Heart Assoc. 2018; 7:e010378. 30371266 10.1161/JAHA.118.010378 PMC6474944

[30] Brinegar A.E., Xia Z., Loehr J.A., Li W., Rodney G.G., Cooper T.A. Extensive alternative splicing transitions during postnatal skeletal muscle development are required for calcium handling functions. Elife. 2017; 6:doi:10.7554/eLife.27192. 10.7554/eLife.27192 PMC5577920 28826478

[31] Konig J., Zarnack K., Rot G., Curk T., Kayikci M., Zupan B., Turner D.J., Luscombe N.M., Ule J. iCLIP reveals the function of hnRNP particles in splicing at individual nucleotide resolution. Nat. Struct. Mol. Biol. 2010; 17:909–915. 20601959 10.1038/nsmb.1838 PMC3000544

[32] Gruber A.J., Schmidt R., Gruber A.R., Martin G., Ghosh S., Belmadani M., Keller W., Zavolan M. A comprehensive analysis of 3′ end sequencing data sets reveals novel polyadenylation signals and the repressive role of heterogeneous ribonucleoprotein C on cleavage and polyadenylation. Genome Res. 2016; 26:1145–1159. 27382025 10.1101/gr.202432.115 PMC4971764

[33] Velusamy T., Shetty P., Bhandary Y.P., Liu M.C., Shetty S. Posttranscriptional regulation of urokinase receptor expression by heterogeneous nuclear ribonuclear protein C. Biochemistry. 2008; 47:6508–6517. 18494499 10.1021/bi702338y

[34] Choi Y.D., Dreyfuss G. Monoclonal antibody characterization of the C proteins of heterogeneous nuclear ribonucleoprotein complexes in vertebrate cells. J. Cell Biol. 1984; 99:1997–1204. 6209285 10.1083/jcb.99.6.1997 PMC2113551

[35] Sureban S.M., Murmu N., Rodriguez P., May R., Maheshwari R., Dieckgraefe B.K., Houchen C.W., Anant S. Functional antagonism between RNA binding proteins HuR and CUGBP2 determines the fate of COX-2 mRNA translation. Gastroenterology. 2007; 132:1055–1065. 17383427 10.1053/j.gastro.2006.12.031

[36] Mukhopadhyay D., Houchen C.W., Kennedy S., Dieckgraefe B.K., Anant S. Coupled mRNA stabilization and translational silencing of cyclooxygenase-2 by a novel RNA binding protein, CUGBP2. Mol. Cell. 2003; 11:113–126. 12535526 10.1016/s1097-2765(03)00012-1

[37] Pervouchine D., Popov Y., Berry A., Borsari B., Frankish A., Guigo R. Integrative transcriptomic analysis suggests new autoregulatory splicing events coupled with nonsense-mediated mRNA decay. Nucleic Acids Res. 2019; 47:5293–5306. 30916337 10.1093/nar/gkz193 PMC6547761

[38] Rossbach O., Hung L.H., Schreiner S., Grishina I., Heiner M., Hui J., Bindereif A. Auto- and cross-regulation of the hnRNP L proteins by alternative splicing. Mol. Cell. Biol. 2009; 29:1442–1451. 19124611 10.1128/MCB.01689-08 PMC2648227

[39] Boutz P.L., Stoilov P., Li Q., Lin C.H., Chawla G., Ostrow K., Shiue L., Ares M. Jr, Black D.L. A post-transcriptional regulatory switch in polypyrimidine tract-binding proteins reprograms alternative splicing in developing neurons. Genes Dev. 2007; 21:1636–1652. 17606642 10.1101/gad.1558107 PMC1899473

[40] Makeyev E.V., Zhang J., Carrasco M.A., Maniatis T. The MicroRNA miR-124 promotes neuronal differentiation by triggering brain-specific alternative pre-mRNA splicing. Mol. Cell. 2007; 27:435–448. 17679093 10.1016/j.molcel.2007.07.015 PMC3139456

[41] Spellman R., Llorian M., Smith C.W. Crossregulation and functional redundancy between the splicing regulator PTB and its paralogs nPTB and ROD1. Mol. Cell. 2007; 27:420–434. 17679092 10.1016/j.molcel.2007.06.016 PMC1940037

