# Supplementary material for: Reciprocal regulation of hnRNP C and CELF2 through translation and transcription tunes splicing activity in T cells
Source: Nucleic Acids Res. 2020 Apr 27;48(10):5710–9. doi: 10.1093/nar/gkaa295 (PMC7261192; doi:10.1093/nar/gkaa295)
Supplement: gkaa295_Supplemental_Files [file gkaa295_supplemental_files.zip › Figures_Supplemental_v2.pdf]

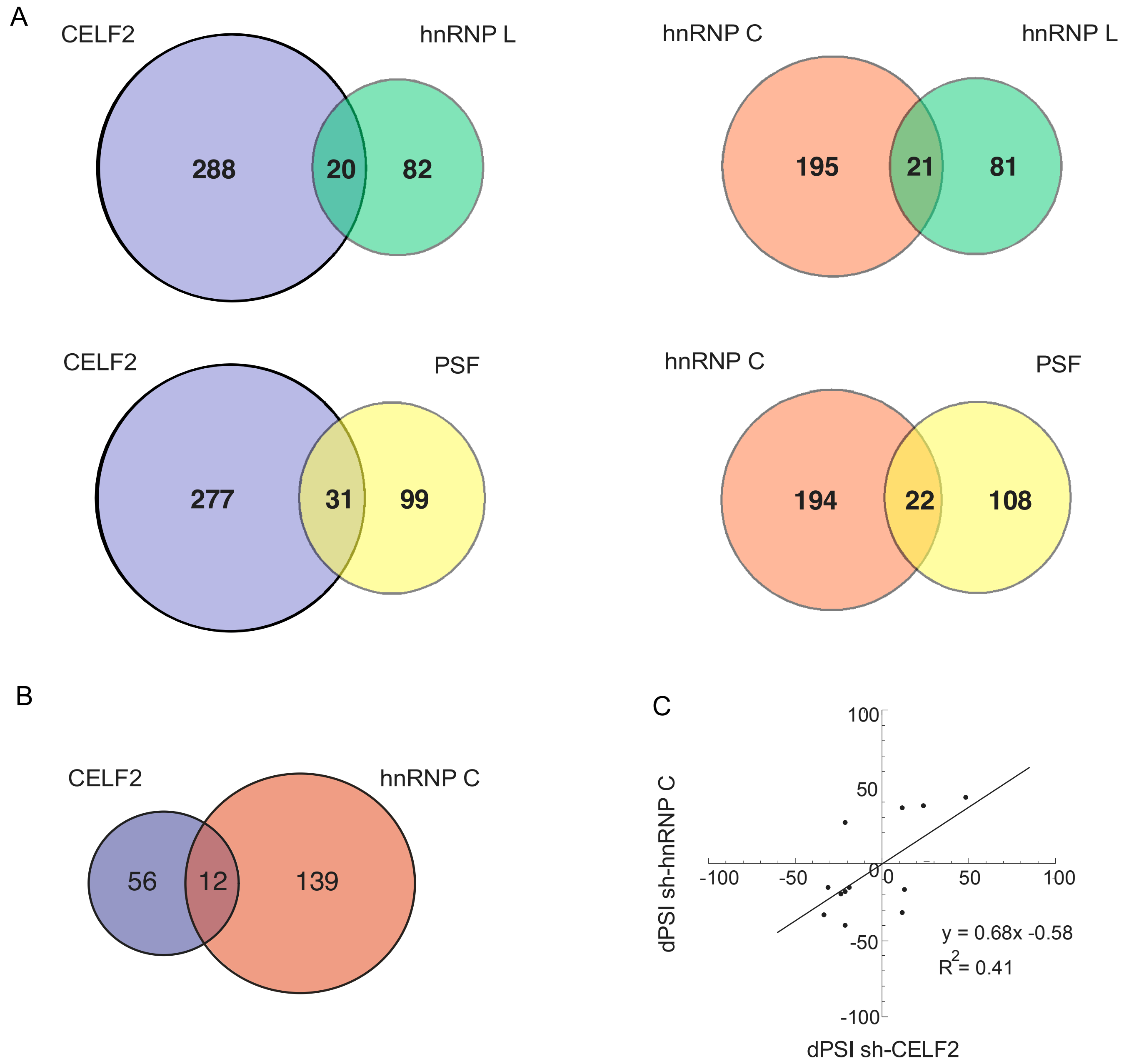

Supplemental Figure 1. Additional analysis of overlap of splicing targets of CELF2 and hnRNP C. (A) Overlap of splicing targets of CELF2 and C compared with those of other hnRNPs also assayed by RASL-Seq in stimulated Jurkat cells. (data from Yarosh et al., 2015; Mallory et al., 2015). (B) Overlap of splicing targets of CELF2 and hnRNP C in unstimulated Jurkat cells. (C) Correlation of ΔPSI of the 12 splicing events sensitive to both shCELF2 and shHNRNPC in unstimulated Jurkat cells.

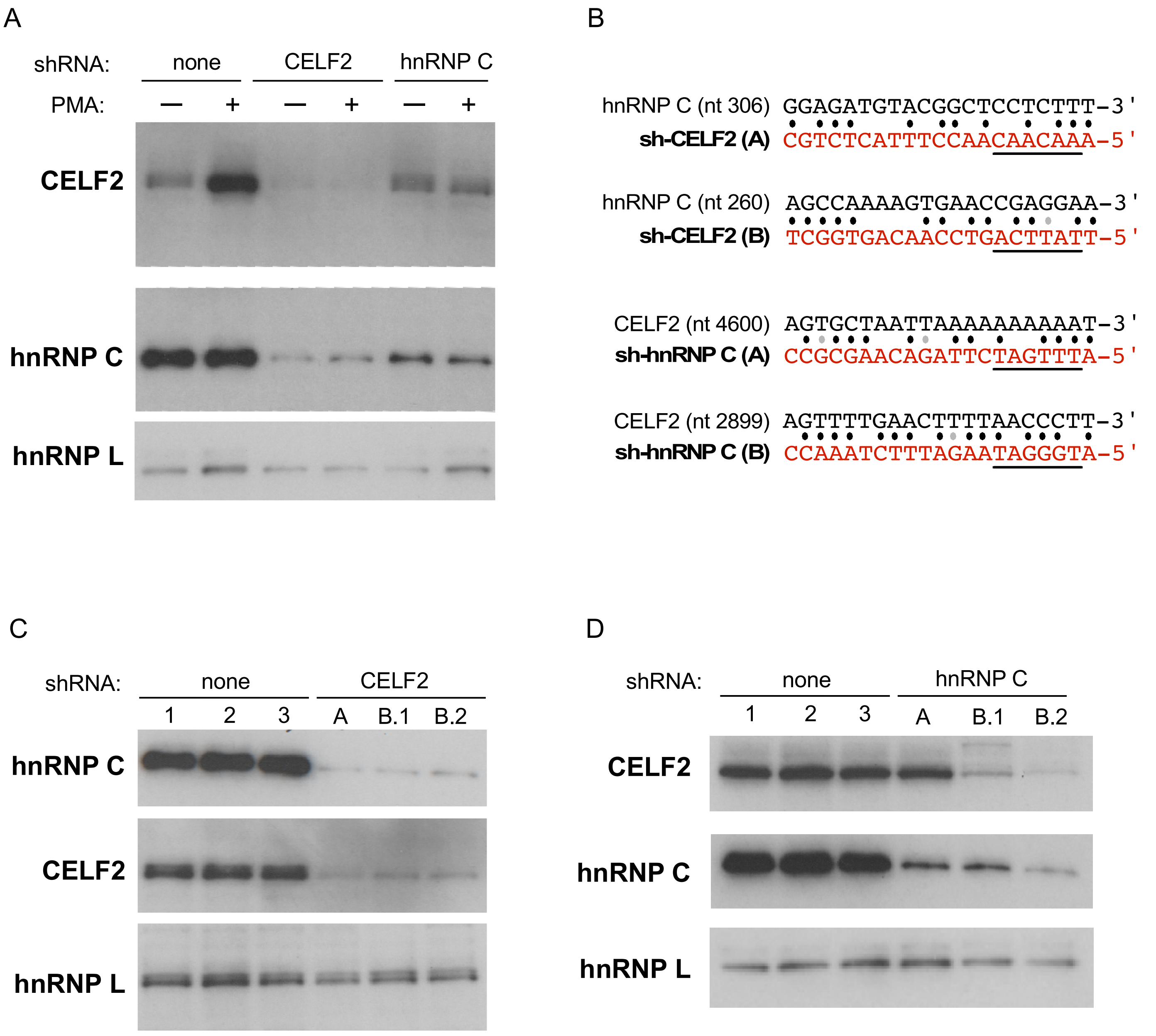

Supplemental Figure 2. Coordinated expression of hnRNP C and CELF2. (A) Expanded Western blot of CELF2 (top panel) or hnRNP C (middle) in cells in which shRNA against CELF2, hnRNP C or mock control was induced for 48 hours. HnRNP L (bottom) is used as a loading control. (B) Best alignment of sh-CELF2 or sh-hnRNP C shRNAs with the other mRNA. Underline indicates location of seed sequence for which complementarity is critical for function. (C) Western blot of hnRNP C, CELFs and hnRNP L in the presence of two CELF2-targeting shRNA sequences (A and B). Two independent clones expressing shRNA B are shown. (D) Western blot of hnRNP C, CELFs and hnRNP L in replicate clones of wildtype Jurkat cells (WT) or in the presence of two hnRNP C-targeting shRNA sequences (A and B). Two independent clones expressing shRNA B are shown

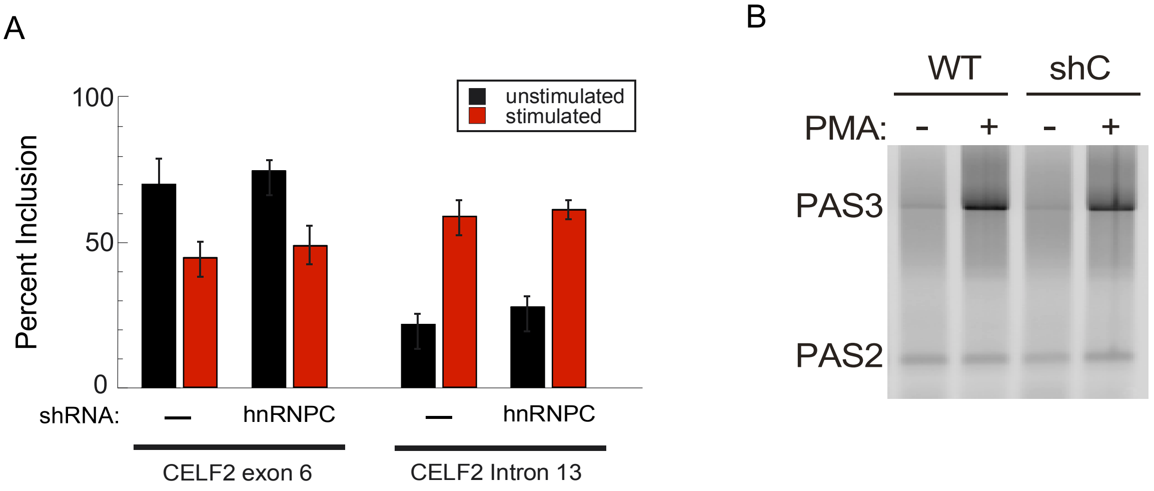

Supplemental Figure 3. Depletion of hnRNP C does not impact splicing or polyadenylation of CELF2. (A) Quantification of inclusion of variable exon 6 or retention of intron 13 in CELF2 mRNA 24 hours after doxycycline induction of shRNA against hnRNP C. Both inclusion of exon 6 and retention of intron 13 have been shown to limit production of full length protein. (B) 3'RACE analysis of polyadenylation site in the 3'UTR of CELF2 24 hours after doxycycline induction of shRNA against hnRNP C (shC). All events assayed here have been shown to be responsive to PMA and to CELF2 autoregulation (Mallory et al., 2015; Ajith et al., 2016; Chatrikhi et al., 2019). Therefore the assays were all done at 12 hours, as hnRNP C is reduced 2-3 fold at this point without any loss of CELF2.

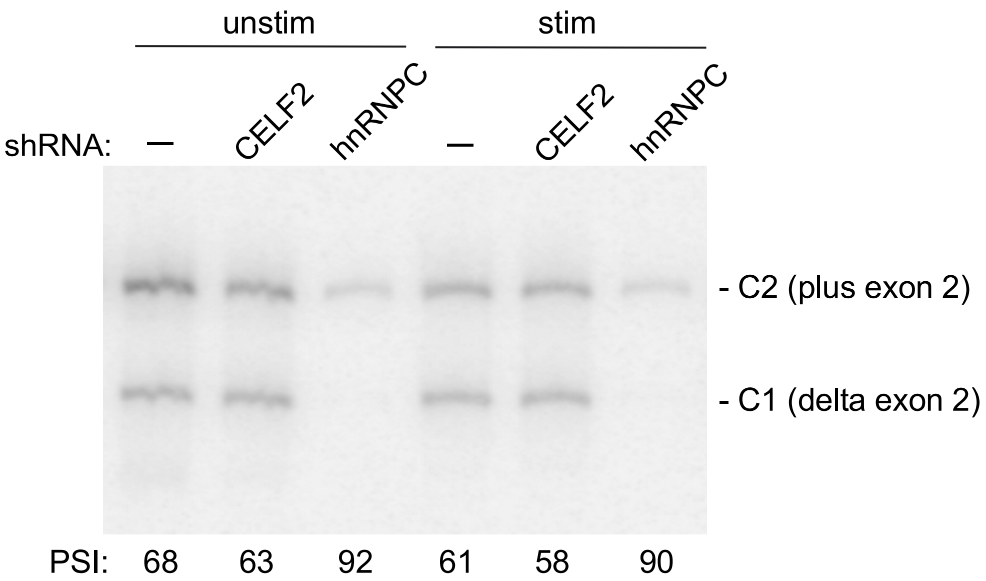

Supplemental Figure 4. Depletion of CELF2 does not alter splicing of hnRNP C. RT-PCR analysis of hnRNP C isoforms C1 and C2, which result from inclusion or skipping of exon 2, in unstimulated or PMA-stimulated Jurkat cells. Depletion of CELF2 has no impact on isoform ratio as quantified as Percent Spliced In (PSI) of exon 2. Depletion of hnRNP C reduces overall mRNA to the point that the C1 isoform is no longer detectable, thus skewing the quantification under these conditions.

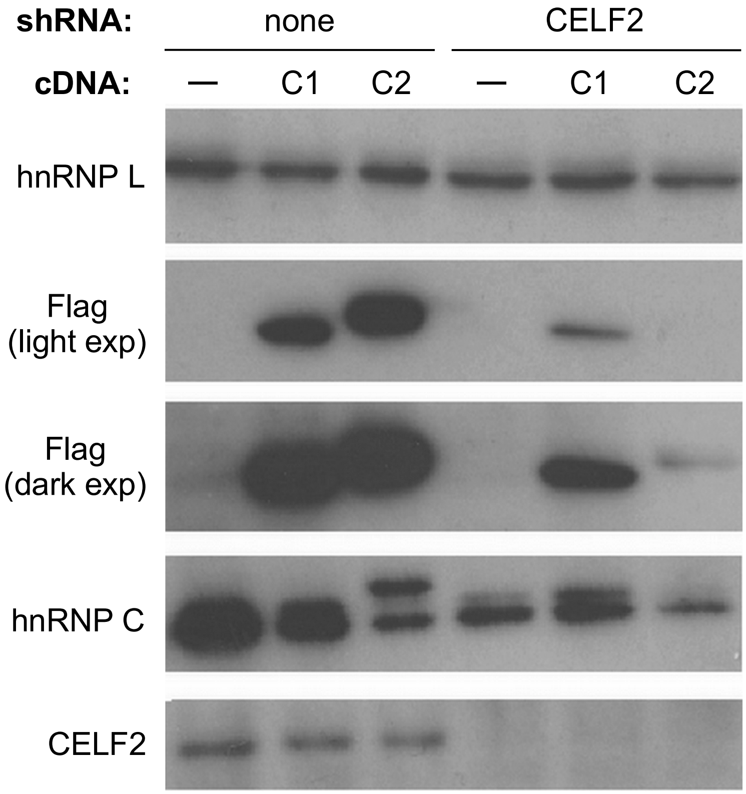

Supplemental Figure 5. Depletion of CELF2 reduces protein expression of cDNA-encoded hnRNP C. Western blot analysis of hnRNP L (loading control), Flag-tagged cDNA-encoded hnRNP C (C1 and C2 isoforms), hnRNP C (endogenous and cDNA-encoded) and CELF2 in Jurkat cells expressing the indicated Flag-tagged hnRNP C cDNA and either lacking (no shRNA) or also expressing CELF2 shRNA.

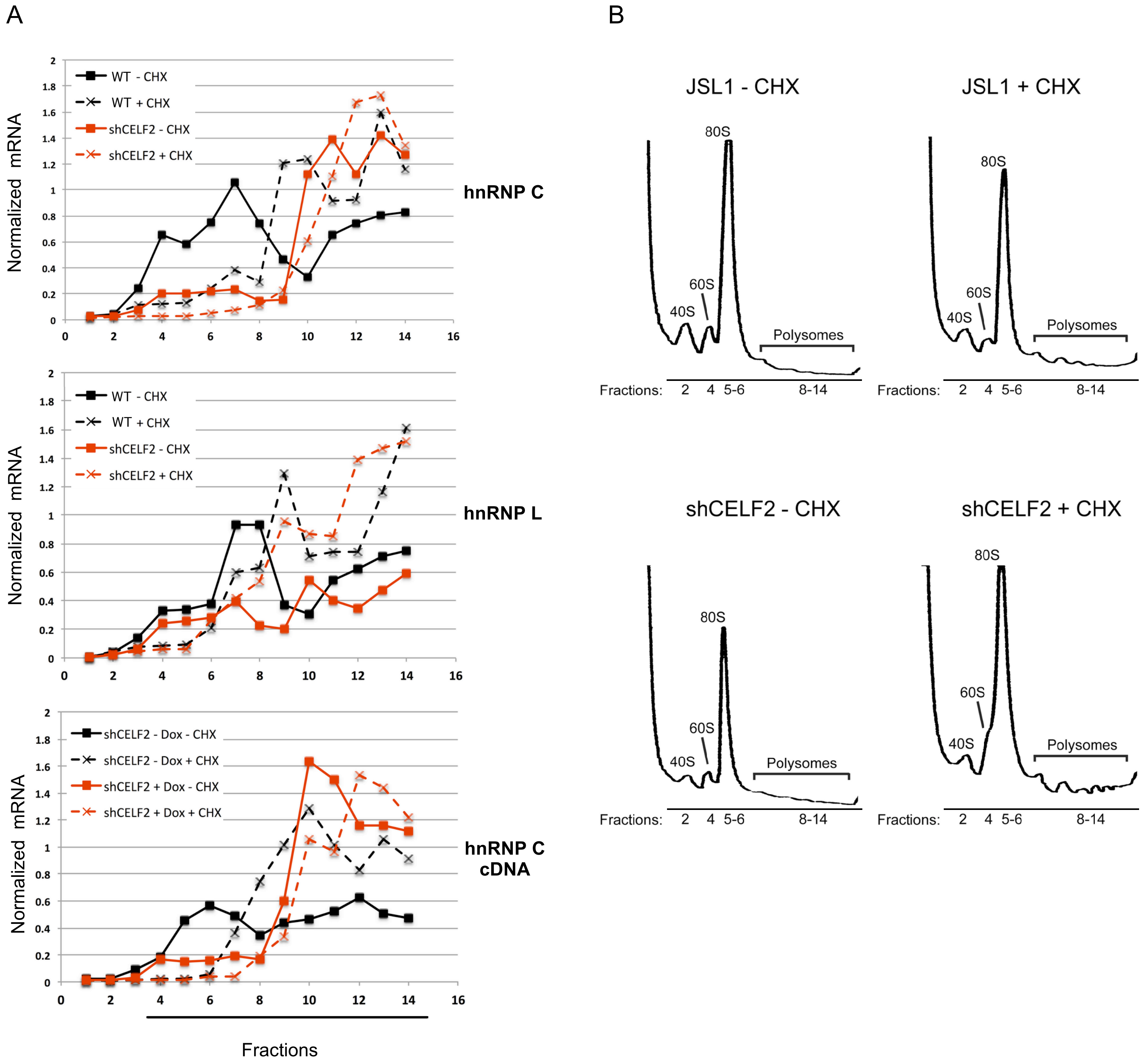

Supplemental Figure 6. Polysome analysis of hnRNP C, hnRNP L and Flag-hnRNP C-cDNA mRNAs. (A) Biologically independent replicate from the experiment shown in Figure 4 C-E. (B) Polysome profiles from all conditions. Note overall reduction in polysomes in the absence of cycloheximide (CHX) consistent with general ribosome run off under these conditions.

A

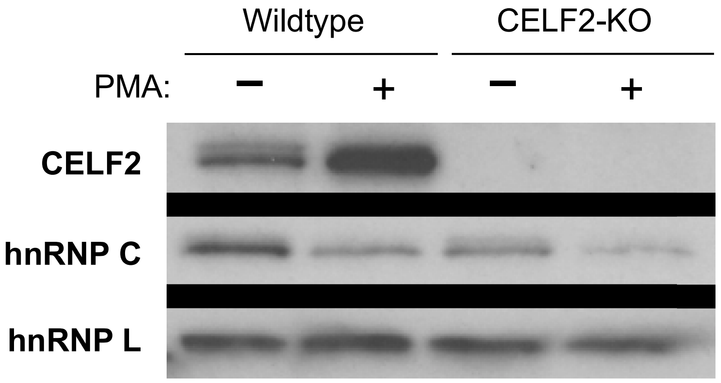

B

RNA-seq expression (FPKM) in Muscle				
Timepoint:	E18	P2	P28	P154
Celf2	19.52	19.31	7.07	9.13
Hnrnpc	74.2	47.65	21.76	19.96

from Brinegar et al., eLife 2017 (ref 30)

Supplemental Figure 7. Coordinated regulation of CELF2 and hnRNP C in T cell activation and muscle development. (A) Western blot of expression of hnRNP C upon stimulation of wildtype Jurkats or those in which CELF2 is knocked out by CRISPR as described previously (Chatrikhi et al., Cell Reports 2019, ref 16). (B) Data from transcriptomic analysis of mouse murine muscle development from the indicated citation. Proteomic data from murine heart muscle from ref 13 and 29 in the main text shows a similar decrease in both CELF2 and hnRNP C, although is not quantiifed in a way that allows direct comparison.

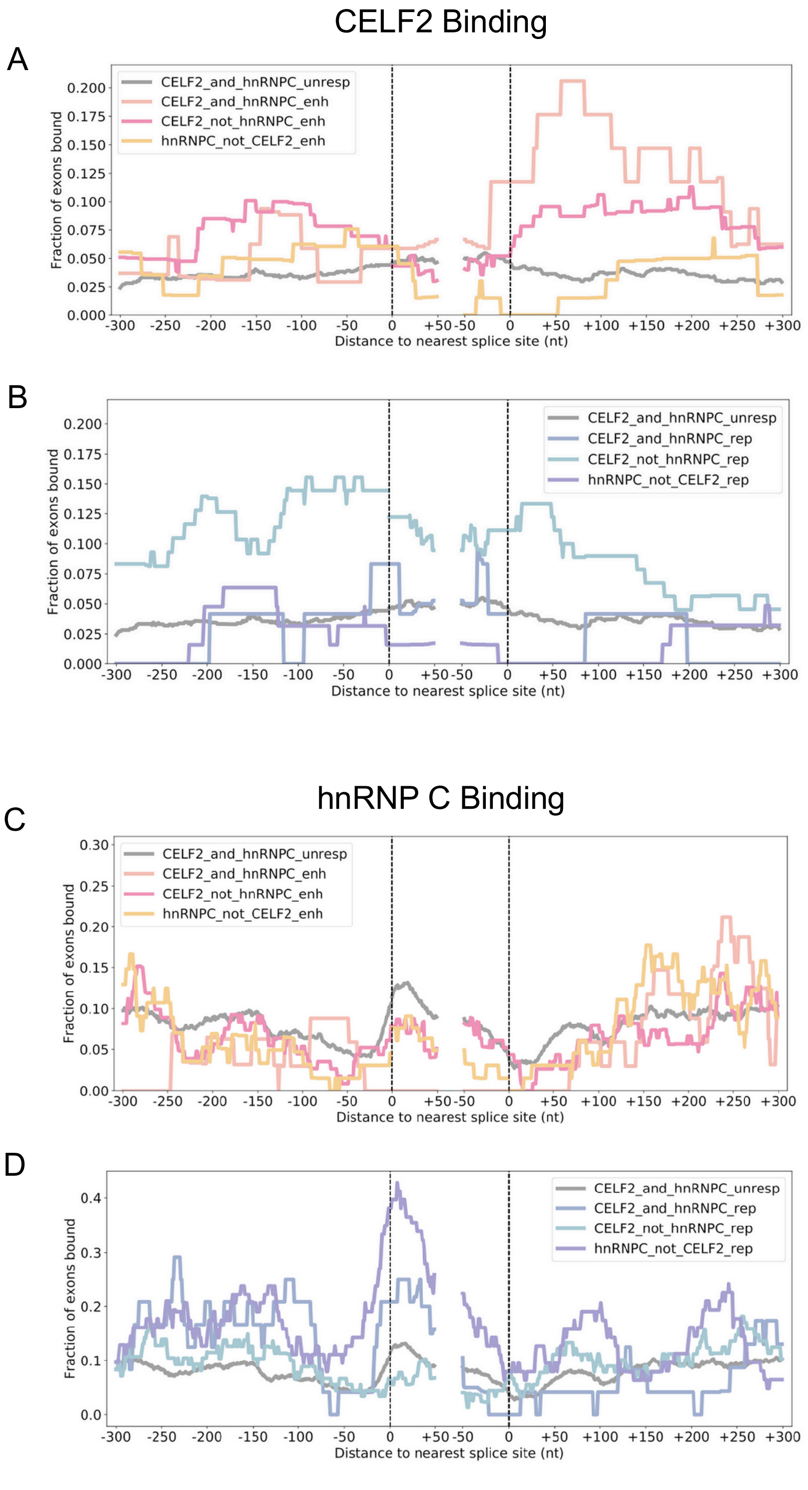

Supplemental Figure 8. CLIP data of CELF2 and hnRNP C reveals binding around regulated exons. Map of CLIP data for CELF2 (panels A and B; from Ajith et al., RNA Biol 2016, ref. 18) or hnRNP C (panels C and D; from https://www.encodeproject.org/) around exons that are preferentially skipped (panels A and C) or included (panels B and D) upon indicated knock-downs. In all cases the gray line indicates binding around unregulated control exons, while colors indicated the various classes of exons as described in the main text. To calculate fraction bound, exons were considered to be bound by the indicated RBP if a significant CLIP peak was present at that position around the exon.

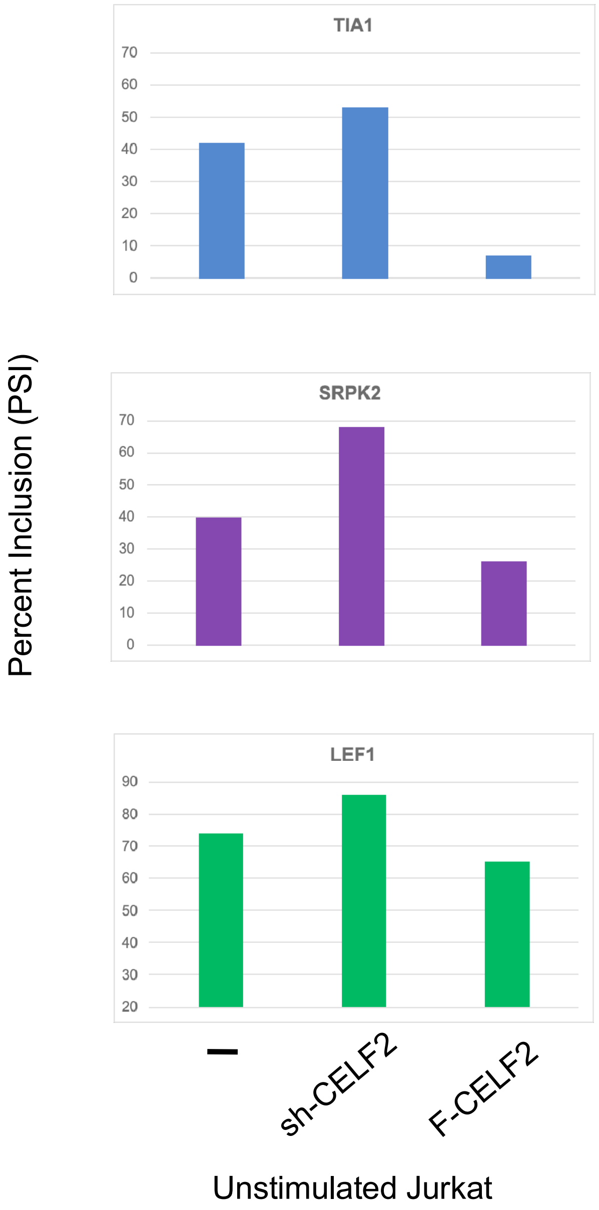

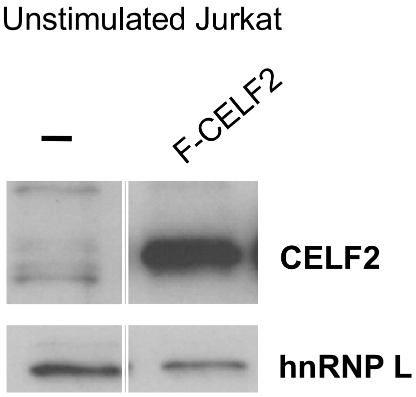

Supplemental Figure 9. Regulation of exon by overexpression of CELF2 Quantification of exon inclusion by RT-PCR upon expression of sh-CELF2 (from main text) or overexpression of flagged-tagged CELF2 (F-CELF2). Exons shown are all predicted to be “CELF2 only” regulation and normally exhibit moderate to high levels of inclusion such that decreased inclusion can be observed. Western blot samples were run on the same gel, cropped to remove un-related lanes.
